# Experimental Study of Almonertinib Crossing the Blood-Brain Barrier in EGFR-Mutant *NSCLC* Brain Metastasis and Spinal Cord Metastasis Models

**DOI:** 10.3389/fphar.2021.750031

**Published:** 2021-09-24

**Authors:** Yuhan Zhang, Yaoshuai Zhang, Wenwen Niu, Xianming Ge, Fuhao Huang, Jinlong Pang, Xian Li, Yu Wang, Wei Gao, Fangtian Fan, Shanshan Li, Hao Liu

**Affiliations:** ^1^ School of Pharmacy, Bengbu Medical College, Bengbu City, China; ^2^ Anhui Province Biochemical Pharmaceutical Engineering Technology Research Center, Bengbu City, China

**Keywords:** almonertinib, non-small cell lung cancer, brain and spinal cord metastases, blood-brain barrier, EGFR-tyrosine kinase inhibitor, transmembrane resistance

## Abstract

Roughly one third of non-small cell lung cancer (NSCLC) patients with epidermal growth factor receptor tyrosine kinase inhibitor (EGFR-TKI)-sensitive mutated (EGFRm) tumors experience disease progression through central nervous system (CNS) metastases during treatment. Although EGFR-TKIs have been reported to be favored in some patients with EGFRm NSCLC CNS metastases, novel EGFR-TKIs with proven efficacy in CNS pathologies are clinically needed.To investigate whether almonertinib, a novel third-generation EGFR-TKI for NSCLC, can cross the blood-brain barrier (BBB) and deliver treatment for EGFR-mutant NSCLC brain metastases and spinal cord metastases, we constructed NSCLC brain metastasis and spinal cord metastasis models *in vivo* to observe the anti-tumor effects of almonertinib. Using ABCB1-MDCK and BCRP-MDCK monolayer cells as the *in vitro* study model, the effects of transport time and drug concentration on the apparent permeability coefficient of almonertinib and its active metabolite, HAS-719, were investigated. The results of this study show that almonertinib can significantly inhibit PC9 brain and spinal cord metastases. Pharmacokinetic studies in mice revealed that almonertinib has good BBB penetration ability, whereas the metabolite HAS-719 does not easily penetrate the BBB. Early clinical evidence of almonertinib activity in patients with EGFRm-advanced NSCLC and brain metastases has also been reported. In conclusion, almonertinib easily penetrates the BBB and inhibits advanced NSCLC brain and spinal cord metastases.

**GRAPHICAL ABSTRACT:**
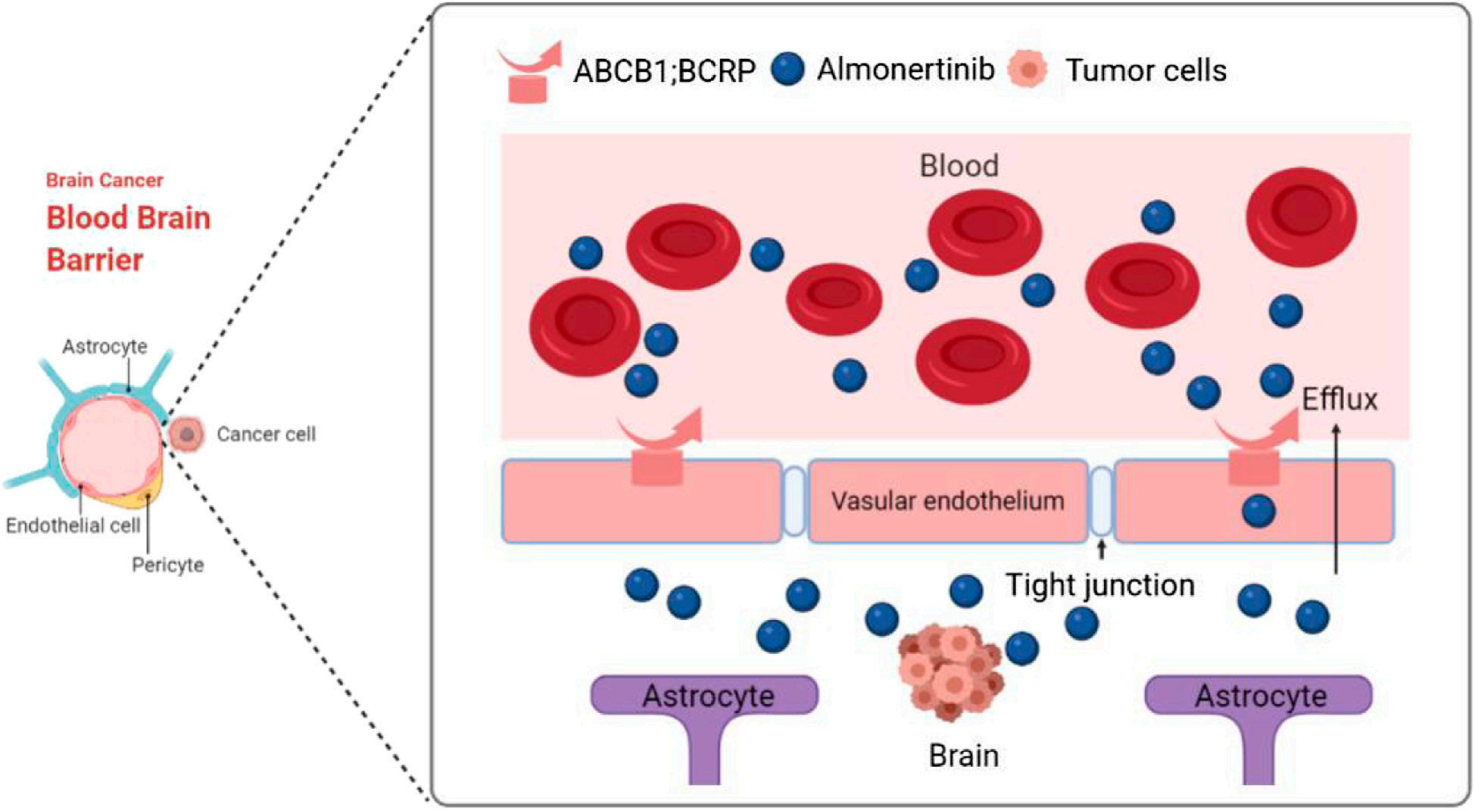
Third-generation EGFR-TKI almonertinib has a low affinity for blood-brain barrier efflux transporters ABCB1 and BCRP, and this can effectively improve the ability of almonertinib to penetrate the brain and successfully treat brain and spinal cord tumors.

## Introduction

According to the data available on the GLOBOCAN 2020 database, lung cancer is still the leading cause of cancer-related deaths. It is estimated that 1.8 million people die of lung cancer worldwide, accounting for 18% of total deaths. Non-small cell lung cancer (NSCLC) accounts for approximately 85% of all lung cancers (Morgensztern et al., 2010). Patients with central nervous system (CNS) metastases, such as brain and spinal cord metastases, have a poor prognosis and poor quality of life. Epidermal growth factor receptor (EGFR) mutations are the most frequent driver mutations in Asian patients with NSCLC. Brain metastases occur in approximately 40% of Asian patients with EGFR mutations before or during treatment (Gow et al., 2009; Luo et al., 2017).

The poor ability of first and second generation EGFR tyrosine kinase inhibitors (TKIs) (e.g., gefitinib, erlotinib, and afatinib) to penetrate the BBB may be due to the influence of ATP binding cassette subfamily B member 1 (ABCB1) and breast cancer resistance protein (BCRP) efflux transporters, which are involved in the removal of toxins, drugs, and chemotherapeutics from the central nervous system (CNS) (Clarke et al., 2010; Jackman et al., 2015). Whole brain radiotherapy (WBRT) has been the standard treatment for patients with brain metastases. However, because WBRT can cause cognitive decline, its role in the treatment of patients with brain metastases remains controversial (Borgelt et al., 1980, 1981). In addition, platinum-based chemotherapeutic drugs generally do not have the ability to penetrate the blood-brain barrier (BBB) to reach intracranial lesions, and therefore, they cannot achieve the desired effect and are not effective against mutations (Sanchez-Covarrubias et al., 2014).

Osimertinib is a third-generation oral, irreversible, selective EGFR TKI that was first approved for a positive EGFR T790M mutation. In the FLAURA study of brain metastasis subgroup, osimertinib, in comparison with the standard treatment, showed statistically and clinically significant results (Leighl et al., 2020). The risk of CNS progression and death was significantly reduced (Reungwetwattana et al., 2018; Soria et al., 2018). However, osimertinib is not an ideal treatment in terms of toxicity. In the AURA3 test (Mok et al., 2017), grade 1 and 2 diarrhea, rashes, and other adverse reactions occurred in 41 and 34% of patients, respectively. Importantly, the use of osimertinib in combination with PD-L1 immune checkpoint inhibitors resulted in a high incidence of interstitial pneumonia, which may be related to the high (∼10%) concentration of AZ5104, the active metabolite of osimertinib in the human body (Mok et al., 2017). Therefore, it is important to develop novel third-generation EGFR inhibitors against EGFR T790 mutations.

Almonertinib is the second third-generation targeted drug for treating lung cancer worldwide, and it has been approved by the China Food and Drug Administration. Structure-activity relationship (SAR) analyses and structural optimization studies have revealed that almonertinib effectively prevents the formation of non-selective active metabolites such as AZ5104. Preliminary clinical data show that almonertinib has good efficacy and safety in the treatment of T790M-positive, locally advanced or metastatic NSCLC (Yang et al., 2020; Nagasaka et al., 2021; Zhang et al., 2021). However, there are only a few reports on the efficacy of almonertinib in brain and spinal cord metastases of NSCLC. In our study, the pharmacokinetics of almonertinib in mice with brain metastases and the anti-tumor efficacy of almonertinib in mice with brain and spinal cord metastases were observed. Using ABCB1-MDCK and BCRP-MDCK cell models (established in this study) that highly express *ABCB1* and *BCRP*, we studied the bidirectional transmembrane transport characteristics of almonertinib and determined whether almonertinib is a substrate of ABCB1 and BCRP and has the ability to penetrate the blood-brain barrier. Furthermore, we discuss the clinical efficacy of almonertinib in the treatment of patients with NSCLC brain and spinal cord metastases.

## Materials and Methods

### Cell Culture and Animals

Madin-Darby canine kidney (MDCK) [NBL-2] (Procell CL-0154) cell line was provided by Procell Life Science & Technology Co., Ltd. PC9 (exon 19 deletion) cells were obtained from Saiku Biotechnology Co., Ltd. (Guangdong, China). PC9 cells and MDCK cells were cultured in RPMI 1640 medium (Gibco, China) and DMEM medium (Gibco, China). The media were supplemented with 10% fetal bovine serum (FBS; Gibco, Australia). All cells were grown in an atmosphere containing 5% CO_2_ at 37°C.

Nude mice were purchased from Changzhou Cavens Laboratory Animal Co., Ltd. (Jiangsu, China) and raised in a specific pathogen-free (SPF) environment. All animal experiments were carried out in accordance with the UK. Animals (Scientific Procedures) Act, 1986 and associated guidelines: the EU Directive 2010/63/EU for animal experiments or the National Institutes of Health Guide for the Care and Use of Laboratory Animals (NIH Publications No. 8023, revised 1978).

### Production of PC9-LUC, ATP Binding Cassette Subfamily B Member 1-Madin-Darby Canine Kidney Cells and Breast Cancer Resistance Protein-Madin-Darby Canine Kidney Cells

To establish an MDCK transgenic cell line that can stably express human ABCB1 and BCRP and to identify whether it is suitable for drug delivery experiments, lentivirus shuttle plasmids pLVX-CMV-ABCB1 and pLVX-CMV-BCRP and their auxiliary packaging plasmids PSPAX2 and PMD2G, were extracted with high purity and free of endotoxin, respectively. 293T cells were transfected with Lipofectamine™ 3000 transfection reagent, and the medium was changed 6 h after transfection. After culturing for 24 and 48 h, the cell supernatants, rich in lentivirus particles, were collected and concentrated by ultracentrifugation. Before lentivirus transfection for 18–24 h, adherent MDCK cells were spread on 24-well plates at a density of 1 × 10^5^/well. The number of cells transfected with lentivirus was approximately 2 × 10^5^/well. The next day, the original medium was replaced with 2 ml of fresh medium containing 6 μg/ml polybrene, and then an appropriate amount of virus suspension; the plates were incubated at 37°C. After 4 h, 2 ml of fresh medium was added to dilute polybrene. The cells were cultured for another 24 h, and the medium containing virus was replaced with fresh medium. After 3–4 days of transfection, puromycin was added to screen the cell lines stably expressing *ABCB1* and *BCRP*.

The cells used in the mouse intracranial tumor model were transfected with luciferase-containing plasmid pGL4.50 (luc2/CMV/Hygro). Stably transfected cell clones were screened using a serial dilution of hygromycin B (10 mg/ml). The fluorescence signal of the cells in the *in vitro* imager (FX-PRO, United States) were then detected.

### Surface Plasmon Resonance

The interaction between analyte (almonertinib and osimertinib) and ligand (EGFR mutant protein) was analyzed at room temperature on an Open surface plasmon resonance (SPR) instrument (Nicoya, Canada). First, according to the standard procedure, a COOH sensor chip was installed on the Open SPR instrument. The buffer was run at the maximum flow rate and exhaust bubbles after reaching the signal baseline. Then, 0.25% SDS was injected and run for 1 min to clean the chip surface. The buffer flow rate was then depressed to 20 µl/min and a sample of 200 µl EDC/NHS (1:1) solution was run for 4 min. Then, the injection instrument of the 200 µl ligand was operated for 4 min. Diluted with activation buffer, Once the ligand-chip interaction was completed, the upper sample mouth was rinsed with buffer and emptied by air. Sample 200 µl Blocking (20 µl/min,4 min) solution, buffer (PBS) rinse the sample ring and empty it by air. The baseline was then observed for 5 min to ensure stability. Finally, different concentrations of analyte were injected separately on the surface of the ligand chip (the concentration is detailed in the experimental results), and the analyte was sampled at 20 µl/min. The binding time of the analyte to the ligand was 240 s and the natural dissociation occurred at 480 s. The 200 µl sample flowed through the chip for 240 s at a constant flow rate of 20 µl/min in each cycle.

The combined compounds can be dissociated from the coating surface by acid and alkali. After each measurement, small molecules and target proteins can be separated by 0.25% SDS. Finally, the kinetic parameters of the binding reactions were calculated and analyzed using Trace Drawer software (Ridgeview Instruments AB, Sweden).

### Anti-Proliferation Assay

The safe toxicity range of almonertinib to MDCK, ABCB1-MDCK, and BCRP-MDCK cells was determined using the CCK8 method. The cells in the logarithmic growth phase were digested with 0.25% EDTA-trypsin, and the complete culture medium was washed and centrifuged at 1,000 rpm for 3 min. The cell density was adjusted to 5 × 10^4^/ml, and they were seeded in a 96-well cell culture plate. The cell suspension (100 μl) was added to each well, and the cells adhered to the wall after 24 h of culture. Almonertinib (100 μl) at different concentrations was added (diluted with high-glucose DMEM). Each sample contained six compound holes. In addition, culture medium containing cells without drugs was used as the control group and culture medium without cells was used as the blank control group. After 3 h of administration and culture, 10 μL of CCK8 was added to each well and incubated at 37°C for 30 min. The absorbance (A) of the samples was determined using an enzyme-labeling instrument at 450 nm.

### ABCB1 and BCRP Substrate Assessment

MDCK, ABCB1-MDCK, and BCRP-MDCK cells were inoculated in Tmur75 culture bottles with high-glucose DMEM. The cells were counted using a cell counter, the cell density was adjusted to 2.0 × 10^5^ cells/ml, and the cells were inoculated in 12-well transwell plates [1.13 cm^2^ insert area, 0.4 µm pore size (Corning Life Sciences, New York, NY, United States)]. Thereafter, 0.5 ml of cell suspension was added to the villous surface of each hole (AP side) and 1.5 ml of blank culture medium was added to the basal surface (BL side). After 24 h of culture in the incubator, the fresh culture medium was changed on both AP and BL sides, and the medium was changed every day for 3–5 days. In this experiment, transmembrane resistance was used to evaluate the compactness and integrity of the cell model. When the transmembrane resistance (TEER) is greater than 300 Ω cm^2^, the cell monolayer is considered dense and intact. The higher the TEER value, the tighter the monolayer connection. The TEER of monolayer cells was calculated using the following formula: TEER = (R - Rb) × A; where, R is the resistance of the well inoculated with cells, Rb is the blank well without cell inoculation, and A is the membrane area of the polycarbonate membrane.

Before the transport experiment, the cell monolayer was washed with blank HBSS solution at 37°C three times, and then placed in a CO_2_ incubator and balanced with HBSS solution for 30 min at 37°C. The transport of the AP→BL side: 0.5 ml of the test solution was added to the AP side as the supply pool, whereas 1.5 ml of blank HBSS solution was added to the BL side as the receiving pool. Before sampling, 0.1 ml of the sample was collected from the BL side in the centrifuge tube, and after sampling, 0.1 ml of blank HBSS, transport sample, was added to the BL side to precipitate protein with methanol, and used for liquid chromatography-tandem mass spectrometry (LC-MS) detection. The transport of the BL→AP side: 1.5 ml of the test solution was added to the BL side as the supply pool, whereas 0.5 ml of blank HBSS solution was added to the AP side as the receiving pool. First, 0.1 ml of the sample was collected on the AP side in the centrifuge tube, and then 0.1 ml of HBSS was added to the AP side after sampling. A transport sample was added to the AP side to precipitate the protein with methanol and used for LC-MS detection.

The protein was precipitated with methanol in 0.1 ml of blank HBSS, transport sample, and used for LC-MS detection. Drug absorption was determined by LC-MS/MS. The effects of transport time and mass concentration on the two-way transport and absorption of almonertinib were investigated.

### Mouse Brain Metastasis Xenograft

The brain metastasis model was established via intracranial injection of tumor cells into 6–8-week-old SPF female BALB/c nude mice. The tumor seeding process, which is an aseptic operation, was carried out on an ultra-clean bench. The tumor cells used in this experiment were PC9-LUC cells, and the concentration of the cell suspension was 1 × 10^8^/ml. Pentobarbital sodium at 50 mg/kg body weight was administered via intraperitoneal injection. The top of the mouse skull was cut along the center of the skull from the ears to the eyes. An area of about 1–2 cm of the skull was then wiped repeatedly with a cotton swab to remove the fascia and expose the cranial sutures. Holes were then drilled 1 mm below the coronal suture of the skull and 2–2.5 mm from the sagittal suture while keeping the mouse head level. The cranial drill was inserted vertically and the drill was operated at low speed to avoid brain damage. Each mouse was then placed on the brain stereotaxic device, the tip of the syringe needle was aligned with the hole on the skull, and 3 μl of cell suspension (3 × 10^5^ cells) was injected via a needle at a depth of 3 mm from the skull. The needle was maintained for 1–2 min following cell injection and then withdrawn. A cotton swab was used to compress the wound and medical adhesive was used to bond the skin wounds. Mice were then placed on a heating blanket and then returned to the animal room for recovery and ongoing nurture. Bioluminescent (BLI) signals were measured weekly using the IVIS Xenogen Imaging system (FX-PRO, United States) to monitor tumor growth. In the mouse brain metastatic tumor model, when the intracranial fluorescence signal of mice reached 2 × 10^7^ photons/s, the effects of long-term once daily administration of almonertinib and osimertinib were observed. The mice were divided into five different treatment groups including the control group, almonertinib (10 or 25 mg/kg), and osimertinib (10 or 25 mg/kg). The BLI signal was measured once a week and the body weight of the mice was recorded.

### Mouse Spinal Cord Metastasis Model

In contrast to the construction of the brain metastasis model, the injection site of the spinal cord metastasis model was 1–1.5 mm below the coronal suture and 2–2.5 mm to the right of the sagittal suture of the skull. Each spinal cord metastasis model mice was injected with 3 μl (4 × 10^5^ cells) cell suspension at a speed of 1 μl/min. In a mouse model of spinal cord metastasis, when the fluorescence signal in the spine of mice reached 2 × 10^7^ photons/s, the effects of long-term once daily administration of almonertinib and osimertinib were observed. The mice were divided into five different treatment groups—the control group, almonertinib (5 or 25 mg/kg), and osimertinib (5 or 25 mg/kg). The BLI signal was measured once a week, and the body weight of the mice was recorded.

### Mouse Pharmacokinetic Studies

When the fluorescence signal of the tumor is greater than 2 × 10^7^ photons/s (about 2–3 weeks), 25 mg/kg body weight of almonertinib was administered orally to female BALB/c nude mice bearing PC9-LUC brain metastasis xenograft. The blood and brain tissues of mice with brain metastases were sampled at 0.5, 1, 2, 4, 8, and 24 h post dosing. The blood samples were obtained by removing the eyeballs of mice. The mice were killed by increasing the CO_2_ concentration. The heads of the mice were taken, the skin was cut to expose the skull, and the eye sockets were clamped with large-pointed tweezers, and the midline of the skull was slightly cut off with ophthalmic scissors. Gradually remove the skull from the bottom to the top. When the whole brain is exposed, use ophthalmic forceps to remove the meninges and blood vessels, and then use a bamboo stick to remove the whole brain from the olfactory bulb. The concentration of almonertinib and HAS-719 was determined by LC-MS.

### Liquid Chromatography-Tandem Mass Spectrometry

Chromatographic column:Thermo Hypersil C18 100 × 2.1 mm, 3 μm; Flow rate: 0.4 ml/min; Water phase: 0.1% formic acid aqueous solution; Organic phase: 0.1% formic acid/acetonitrile; Needle wash: methanol; Column oven temperature: 40°C; Autosampler temperature: 10.0°C; Automatic sampling Injection volume of the detector: 5.00 µl.

Ion source: electrospray ionization source; Scanning method: positive ion scanning; Detection method: selective reaction monitoring; Electrospray voltage: (+) 4,000 V; Capillary temperature: 300°C; Atomization temperature: 350°C; Sheath gas: nitrogen (Purity ≥99.999%), 30 Arb; Aux Gas Pressure: Nitrogen (purity ≥99.999%), 10 Arb; Collision gas: high-purity argon (purity ≥99.999%); Data collection time: 5.00 min; Accurately weigh almonertinib and HAS-719 standard products respectively, dissolve them in methanol, and prepare a stock solution of 2.00 mg/ml. Dilute the 2.00 mg/ml stock solution to obtain standard curve solutions with concentrations of 500, 200, 50, 2, 1, and 0.5 ng/ml.

Dissolve the serum sample at room temperature, take 50 μl, add 150 μl methanol, vortex for 5 min, place in a centrifuge at 15,000 rpm for 10 min, take the supernatant for analysis. Weigh the tissue sample, add 2 ml of methanol, grind for 5 min, vortex and mix for 5 min, place it in a centrifuge at 15,000 rpm for 10 min, and take the supernatant for analysis.

The chromatogram collection and integration of almonertinib and HAS-719 are processed by the software Xcilabur 3.0 (Thermo), and linear regression is performed with weighting coefficients.

### Clinical Case Study

Herein, we present details of a clinical case study. In this case, brain metastases were recorded at the beginning of the study, and almonertinib was administered for several weeks to allow magnetic resonance imaging of the brain to observe the progression of the disease. Written informed consent was obtained from the patient. The work has been carried out in accordance with The Code of Ethics of the World Medical Association (Declaration of Helsinki).

### Statistical Analysis

All data are presented as mean ± SEM. All experiments were independently repeated at least three times. Different groups were compared using an unpaired Student’s *t*-test or one-way ANOVA followed by Tukey’s *post-hoc* test. Differences were considered statistically significant at *p* < 0.05. GraphPad Prism 7 software (GraphPad Software, San Diego, CA, United States) was used for statistical analyses.

## Results

### Interaction Between Almonertinib and EGFR-T790 Mutant Protein

**FIGURE 1:**
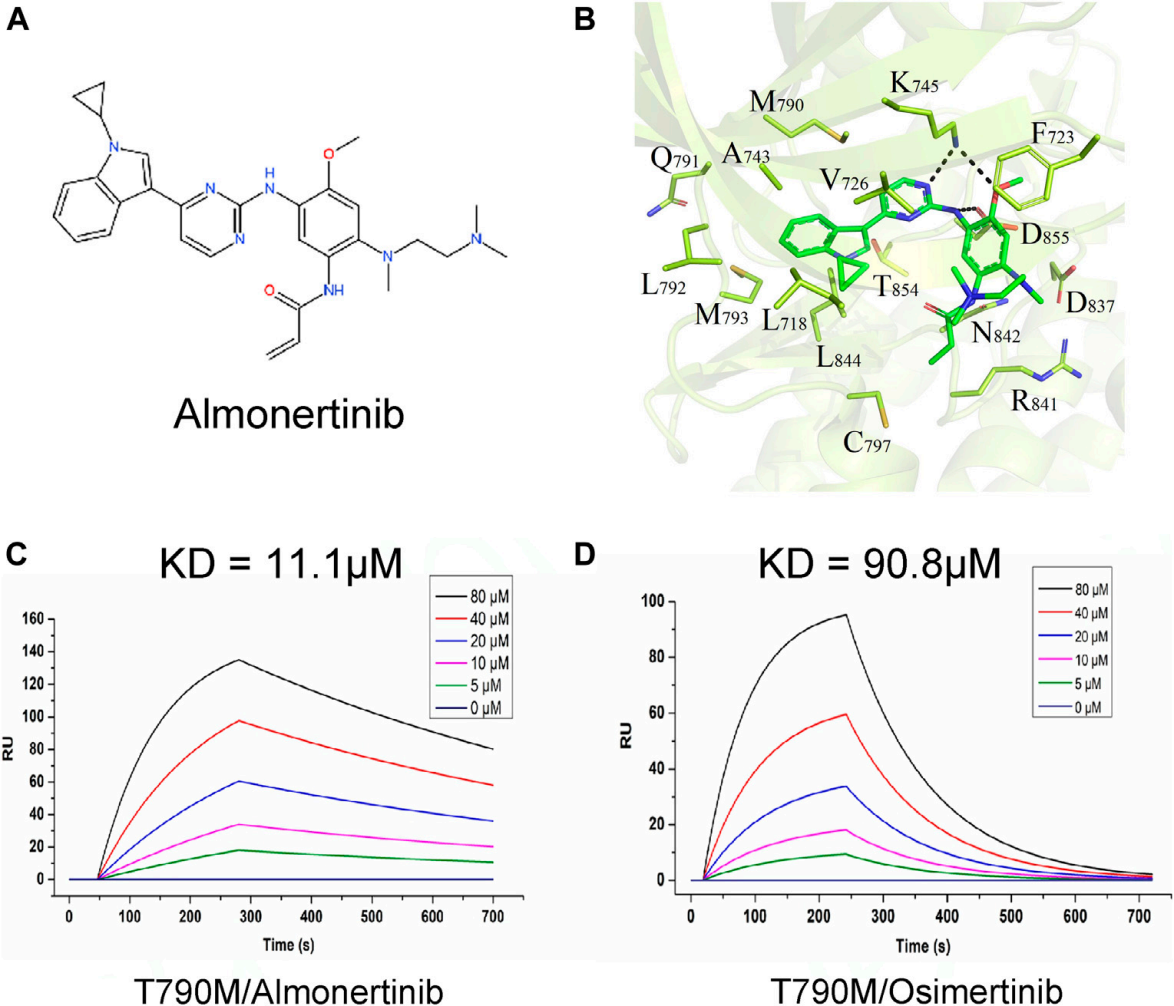
Interaction between almonertinib and EGFR-T790 mutant protein. **(A)** Chemical structure of almonertinib; **(B)** molecular model diagram of interaction between almonertinib and EGFR T790M mutation; **(C)** determination of the affinity of EGFR T790M and almonertinib using the SPR method, the affinity constant was 11.1 μM; **(D)** the affinity of EGFR T790M and osimertinib was 90.8 μM.

To study the interaction between almonertinib and EGFR-T790M protein, their binding mode and affinity were predicted by molecular docking and surface plasmon resonance (SPR). The molecular docking results showed that almonertinib can flexibly bind to small molecule pockets on the EGFR-T790 mutant protein with a better geometrical match. The binding sites of almonertinib and the EGFR-T790 mutant protein are relatively conservative. Almonertinib has a large chemical group and a pure hydrophobic chain. It can form a hydrophobic binding match with protein-binding pockets (e.g., F723, V726, M790, A743, L792, and L844). The other end of the almonertinib branch contains an N-(CH_3_)_2_ group and an -NH-C(=O)- group, which can be inserted into the small groove of the binding cavity, forming a polar bond with N842, D837, and D855. The N atom on the plane ring in the small molecule can form a polar hydrogen bond with K745. Hinges in enzyme proteins often form hydrogen bonds with the corresponding molecules, which are relatively conserved in kinase proteins. However, the amino acid group at the hinge of our kinase protein mutation system does not form conventional hydrogen bonds with small molecules. The polar effect weakens the binding force of this part of the effect. Almonertinib can flexibly bind to the small molecule pocket on the EGFR-T790M mutant protein with good geometric matching, and the binding energy between the two is −7.03 kcal/mol (Figures 1A,B). At the same time, almonertinib showed a higher affinity for T790M (KD = 11.1 µM) than for osimertinib (KD = 90.8 µM) (Figures 1C,D).

### Almonertinib Shows Superior Tumor-Regressing Efficacy in an Epidermal Growth Factor Receptor Mutant Brain Metastases Model

**FIGURE 2:**
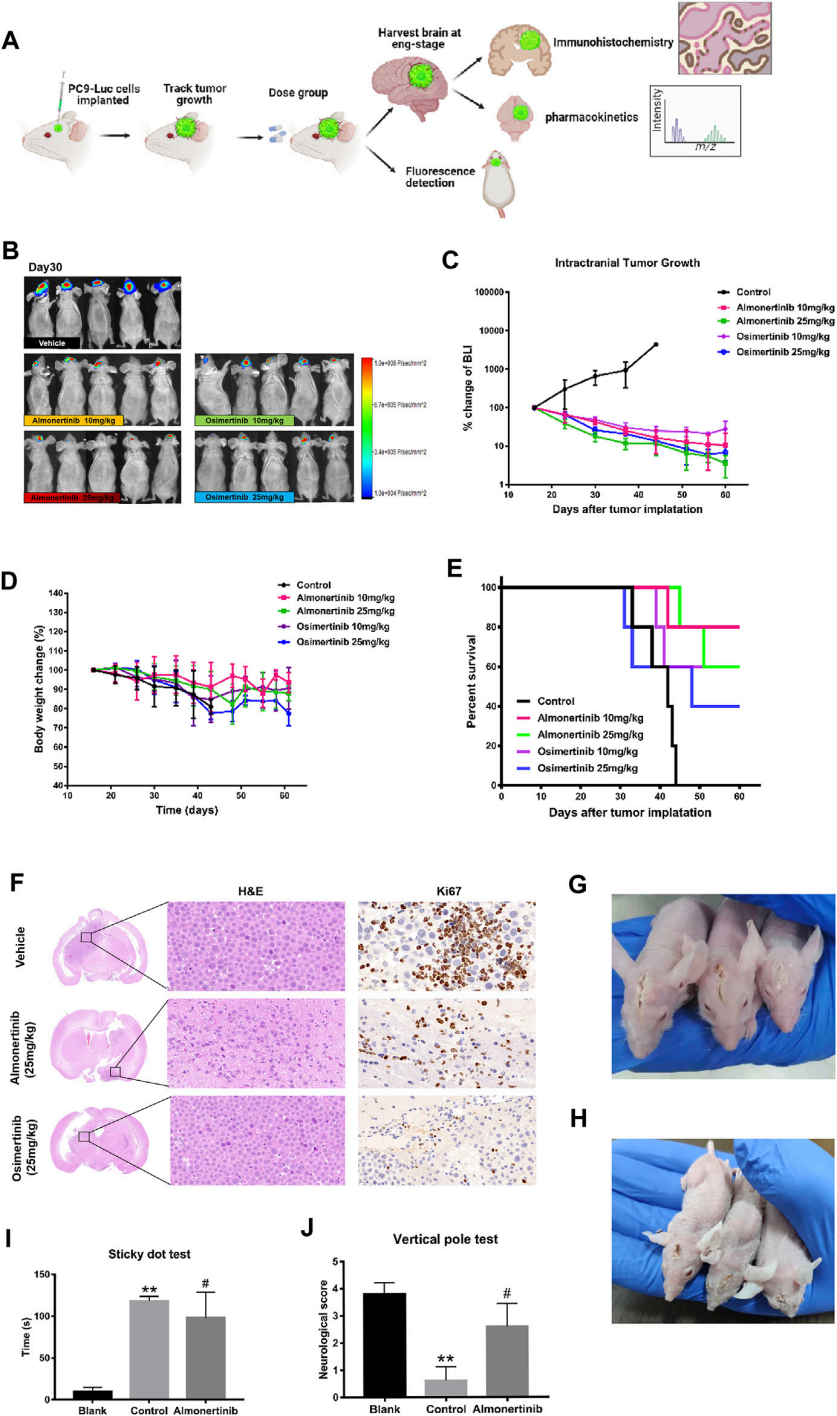
Anti-tumor effect of almonertinib in PC9-LUC brain metastatic tumor model. **(A)** Establishment of a brain metastatic tumor model of non-small cell lung cancer; **(B)** Two weeks after the injection of PC9-LUC, the animals were treated with almonertinib or osimertinib once daily. Almonertinib 10 and 25 mg/kg (Qd) and osimertinib 10 and 25 mg/kg (Qd) were used to treat PC9-LUC brain metastatic tumor mice. Tumor bioluminescence **(C)**, body weight **(D)**, and overall survival rate **(E)** were observed. **(F)** The expression of Ki-67 in the brain tissue was detected by hematoxylin-eosin and immunohistochemical staining. **(G,H)** Images of brain metastases in mice after administration; **(I,J)** neurological score of mice with brain metastases. Scale bar, 50 μm. Data are presented as mean ± SEM (*n* = 5/group). **p < 0.01 vs. vehicle control.

To evaluate the efficacy of almonertinib in inhibiting intracranial tumor growth compared with osimertinib, 3 × 10^5^ PC9-LUC cells were inoculated with intracranial tumors at a position 1 mm from the coronal suture of the skull and 2–2.5 mm to the right of the sagittal suture in female BALB/C mice. The tumor-bearing mice were treated daily with the control, 10 mg/kg almonertinib, 25 mg/kg almonertinib, 10 mg/kg osimertinib, or 25 mg/kg osimertinib. After 2–3 weeks, the bioluminescence signal of the mice was obvious. The daily dose of 25 mg/kg osimertinib in nude mice provides the exposure level was comparable to the daily dose of 80 mg in humans. In this experiment, 10 and 25 mg/kg almonertinib and osimertinib effectively inhibited the growth of intracranial tumors (Figures 2A–C). The body weight of mice treated with both almonertinib and osimertinib decreased after administration. The weight loss in the 25 mg/kg osimertinib dose group was more obvious, and the skin of mice in the osimertinib 25 mg/kg group appeared to be keratinized (Figures 2D, G,H). To evaluate whether the efficacy of almonertinib in the treatment of intracranial tumors was related to the survival rate of mice, we calculated the survival of mice in the control, almonertinib (10 and 25 mg/kg), and osimertinib (10 and 25 mg/kg) groups for 60 days (Figure 2E). The results showed that the overall survival time of mice in the almonertinib group was longer than that of mice in the osimertinib group, and the quality of life achieved with 10 mg/kg almonertinib and 10 mg/kg osimertinib was better than that with 25 mg/kg almonertinib and 25 mg/kg osimertinib. At the same time, the experimental sample size was small and the individual differences of mice were large; therefore, more samples are needed for comparison. Histological analysis showed that the number of proliferative cells significantly decreased in almonertinib-treated mice (Figure 2F). Correspondingly, we performed a sticky dot test and a vertical test on mice to score neurological function and to evaluate motor and sensory nerve function. The mice with brain metastasis in the almonertinib 25 mg/kg group showed a significant improvement in neurological function compared with those in the control group (Figures 2I,J).

### Almonertinib Shows Superior Tumor-Regressing Efficacy in an Epidermal Growth Factor Receptor Mutant Spinal Cord Metastases Model

**FIGURE 3:**
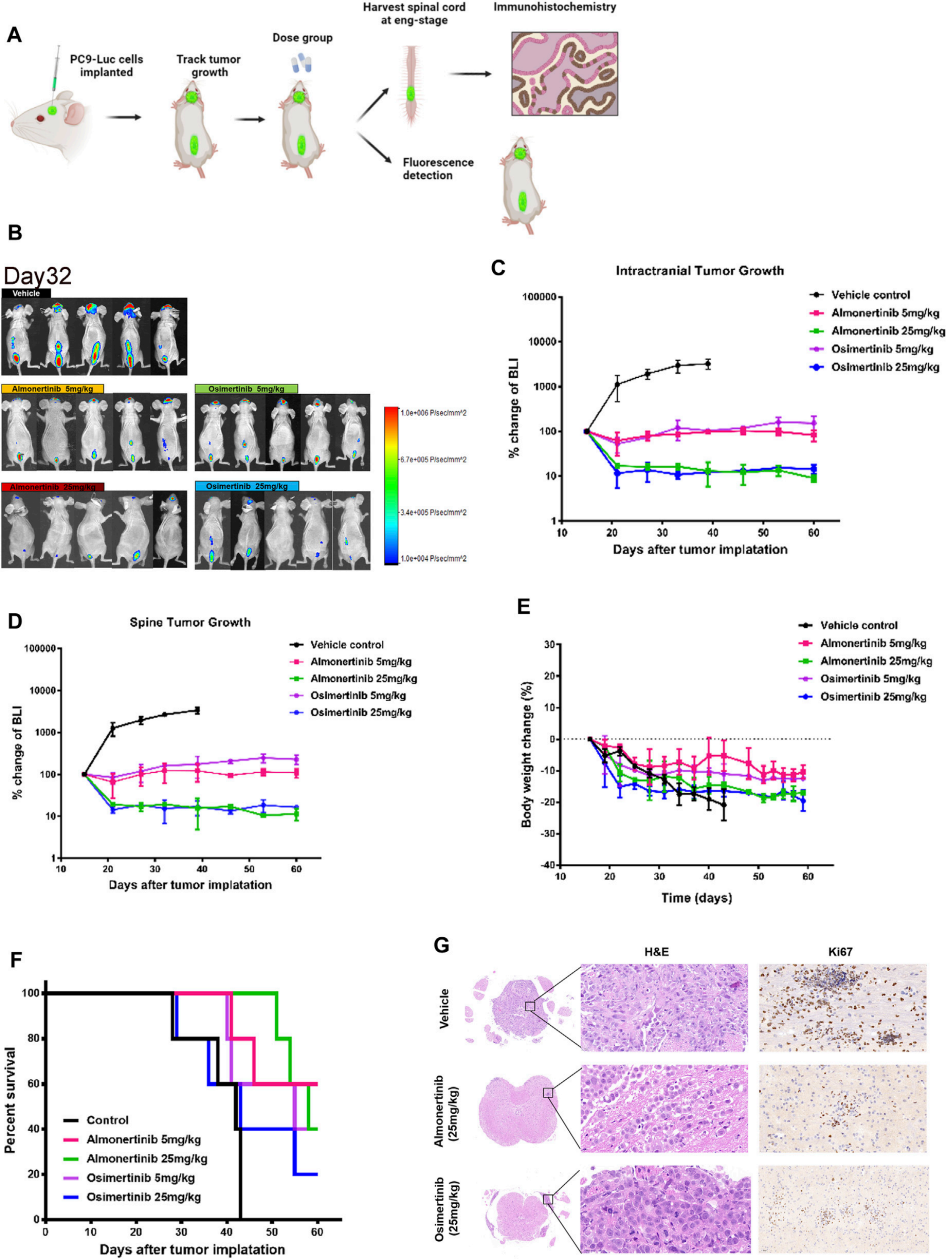
Antitumor effect of almonertinib in PC9-LUC-injected mice with spinal cord metastases. **(A)** Establishment of a spinal cord metastasis model of non-small cell lung cancer with brain metastasis; **(B)** Two weeks after the injection of PC9-LUC, the animals were treated with almonertinib or osimertinib once daily. Almonertinib 5 and 25 mg/kg (Qd) and osimertinib 5 and 25 mg/kg (Qd) were used to treat PC9-LUC-injected mice with brain metastases and spinal cord metastases. Intracranial tumor bioluminescence **(C)**, spinal cord tumor bioluminescence **(D)**, body weight **(E)**, and overall survival rate **(F)** were observed. The expression of Ki-67 in the spinal cord tissue was detected by hematoxylin-eosin staining and immunohistochemical staining. Scale bar, 50 μm. Data are presented as mean ± SEM (*n* = 5/group). **p < 0.01 vs. vehicle control. **(G)** The expression of Ki-67 in the spinal cord tissue was detected by hematoxylin-eosin staining and immunohistochemical staining.

Spinal cord metastasis remains a major problem in NSCLC. To verify whether almonertinib has an inhibitory effect on spine metastasis, we established an NSCLC spinal cord metastasis model in nude mice. For the spinal cord metastasis model, PC9-LUC cells were injected into the brain ventricles of nude mice, and the mice were placed in an SPF environment. After 2–3 weeks, the spinal cord bioluminescence signal of the mice was obvious. These mice were then randomly divided into five groups (five in each group) to receive various treatments. After 16 days of modeling, bioluminescence imaging was used to assess the growth and metastasis of PC9-LUC cells every week. Compared with the control group, the biofluorescence signal of the high-dose almonertinib group and the high-dose osimertinib group was significantly reduced, showing inhibited growth and metastasis of intracranial and spinal cord tumor cells. The biofluorescence signal of the almonertinib and osimertinib low-dose groups did not change significantly compared with that of the control group, and the growth of tumor cells was the same as that before administration (Figures 3A–D). Similar to the treatment of brain metastases, we observed changes in the body weight and survival curve of mice; the weight change in nude mice in the high-dose group was higher than that in the nude mice in the low-dose group (Figures 3E,F). Nude mice in the almonertinib-administered group presented a longer survival time than those in the osimertinib-administered group. After treatment, the spinal cords of the high-dose almonertinib and control mice were dissected for H&E and Ki-67 staining. The high-dose almonertinib group showed a significant inhibition in the proliferation of tumor cells (Figure 3G). In summary, almonertinib can inhibit NSCL brain and spinal cord metastases *in vivo*.

### Almonertinb can Cross the Blood-Brain Barrier and Enter the Brain to Distribute in the Tumor Tissue

**FIGURE 4:**
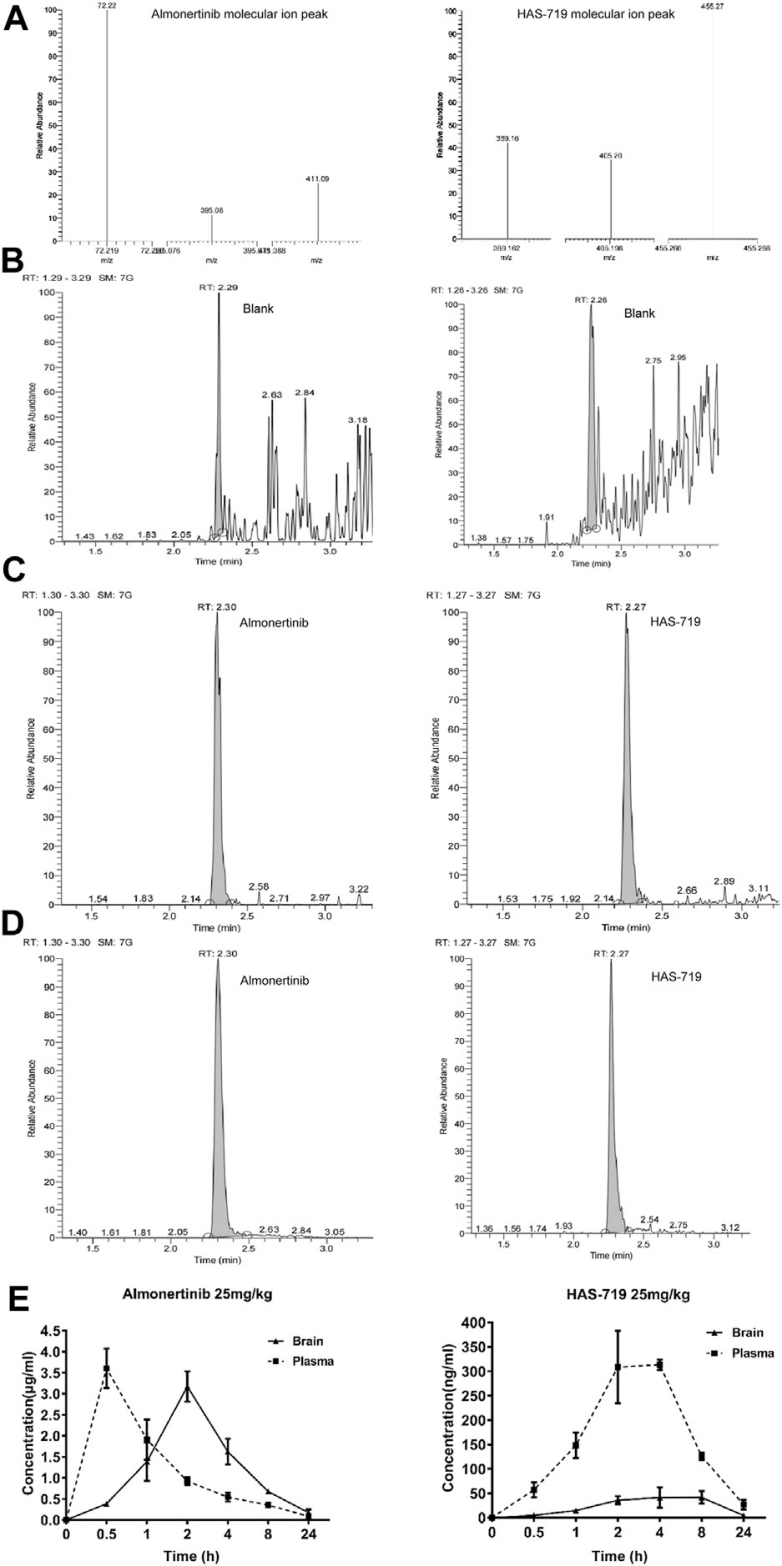
Almonertinib and its metabolite HAS-719 in the brain tissue and plasma of mice with brain metastases were determined by liquid chromatography-mass spectrometry (LC-MS). **(A)** The second-order mass spectra of almonertinib and HAS-7A9; the typical LC-MS chromatograms of almonertinib **(left)** and HAS-7199 **(right)**. **(B)** Blank plasma; **(C)** blank plasma mixed with standard almonertinib and HAS-719; **(D)** almonertinib 25 mg/kg was orally administered to mice. **(E)** Concentration-time profiles of almonertinib and HAS-719 after the oral administration of almonertinib in mice with brain metastases (*n* = 5).

To verify whether almonertinib can penetrate the BBB, the drug concentration in the brain tissue and plasma of mice with brain metastases within 24 h after administration was detected. In the PC9-LUC BM xenograft model, almonertinib demonstrated excellent BBB penetration. The maximum concentration of the compound was detected in the brain at 4 h after a single dose of 25 mg/kg. The blood concentration-time curve of almonertinib and HAS-719 measured by oral administration of almonertinib and HAS-719 in mice with brain metastases was 25 mg/kg. According to the curve of blood concentration at each time point, the plasma concentration of almonertinib increased rapidly within 0.5 h after oral administration, decreased rapidly after 1 h, and reached a maximum at 4 h. Almonertinib could still be detected in the plasma and brain tissue at 24 h. At the same time, we observed that the concentration of almonertinib metabolite HAS-719 in the brain tissue was considerably lower than that in the plasma (Figures 4A–E; Table1).

**TABLE 1:** Pharmacokinetics of almonertinib and HAS-719 in plasma and brain following oral administration of almonertinib at 25 mg/kg to female BALB/c nude mice bearing PC9-LUC tumor xenografts.

Test compound	Dose (mg/kg)	25	
	Tissue	Plasma	Brain
Almonertinib	C_max_ (μmol/L)	6.41	5.36
	tmax (h)	0.5	2
	AUC_0–t_ (μmol/L.h/L)	16.86	30.6
	Tissue/plasma AUC ratio	NA	1.82
	MRT_Last_ (h)	5.42	6.19
HAS-719	C_max_ (μmol/L)	0.65	0.11
	tmax (h)	4	6
	AUC_0–t_ (μmol/L.h/L)	4.96	1.06
	Tissue/plasma AUC ratio	NA	0.21
	MRT_Last_ (h)	6.67	7.44

AUC, area under the plasma or tissue concentration-time curve; AUC_0–t_, area under the plasma or tissue concentration-time curve from time 0 to time t; C_max_, maximum plasma concentration; NA, not applicable; t_max_, time to C_max_; MRT_last_, retention time of the drug in the body.

### Almonertinib is a Substrate of ATP Binding Cassette Subfamily B Member 1 and Breast Cancer Resistance Protein Transporters

**FIGURE 5:**
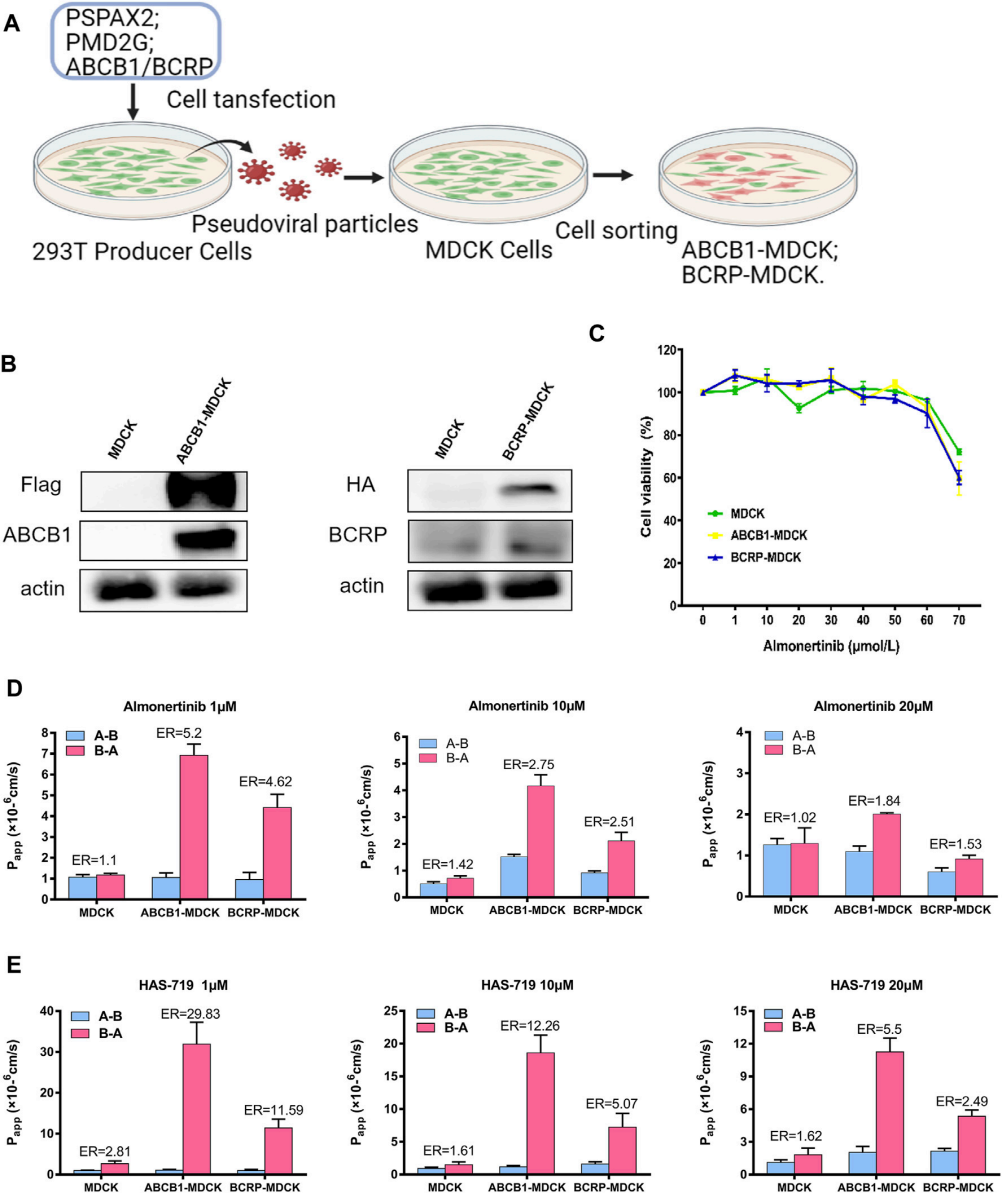
Permeability of almonertinib and HAS-719 across MDCK, ABCB1-MDCK and BCRP-MDCK cell monolayers. **(A)** The production of ABCB1-MDCK and BCRP-MDCK cells. **(B)** The ABCB1-overexpressing and BCRP-overexpressing MDCK cells were identified by western blotting. **(C)** The effects of almonertinib on the viability of MDCK, ABCB1-MDCK, and BCRP-MDCK cells. These cells were treated with almonertinib at a gradient concentration (0–70 μM) for 4 h. **(D)** Permeability of almonertinib (1, 10, and 20 μM), **(E)** HAS-719 (1, 10, and 20 μM) across MDCK, ABCB1-MDCK, and BCRP-MDCK cell monolayers.

There are a series of expatriate transporters on the BBB, and they can further restrict the entry of therapeutic drugs into the brain. Among all expatriate transporters expressed on the BBB, two (ABCB1 and BCRP) are mainly responsible for transporting anticancer drugs that penetrate the brain parenchyma, and back to the circulating blood. To verify whether almonertinib is a substrate of efflux transporters such as ABCB1 and BCRP, we measured the net efflux ratio (ER) of ABCB1 or BCRP at different doses of almonertinib and HAS-719 (Figure 5). Based on the positive substrate criteria of the efflux ratio (ER >2) and the finding of a previous study (Ballard et al., 2016), the relative efflux ratios of almonertinib or its metabolite HAS-719 between ABCB1-MDCK and normal MDCK cells and between BCRP-MDCK and MDCK cells suggest that these agents are ABCB1 and BCRP transporter substrates. In ABCB1-MDCK cells, the efflux ratio of almonertinib dosed at 1 µM was 5.2, at 10 µM was 2.75, and at 20 µM was 1.84. ER in BCRP-MDCK cells at 1 µM was 4.62, at 10 µM was 2.51, and at 20 µM was 1.53.

### Almonertinib Demonstrates Potent Anti-Tumor Activity in a Patient With Epidermal Growth Factor Receptor Mutant Non-Small Cell Lung Cancer

**FIGURE 6:**
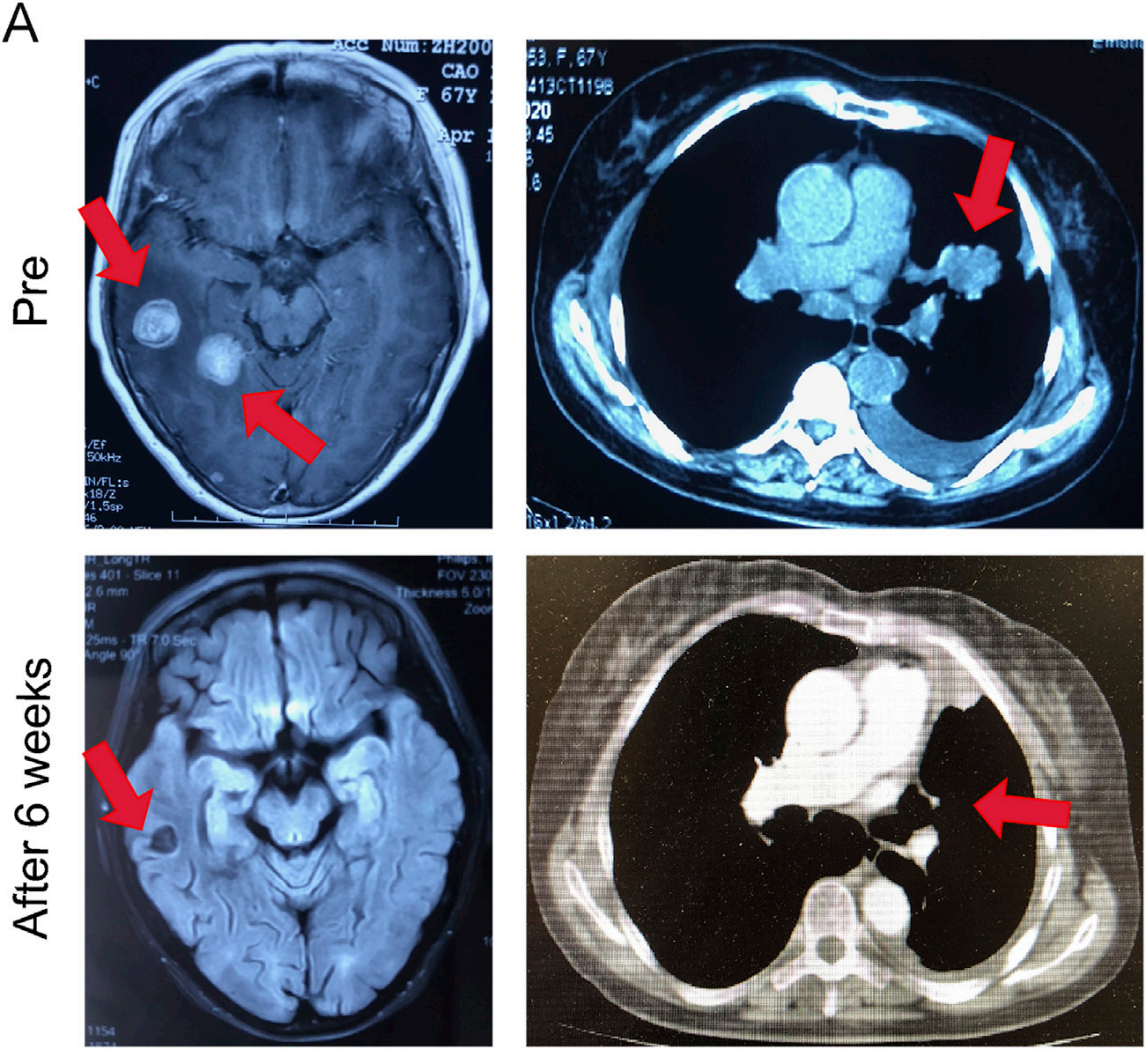
Almonertinib demonstrated potent anti-tumor activity in a patient with EGFR mutant NSCLC. Intracranial and extracranial response after treatment with 110 mg almonertinib in a 67-year old female patient with the EGFR mutation.

A 67-year-old Asian woman with EGFR 19del mutant lung adenocarcinoma and brain metastases was treated with almonertinib 110 mg po qd as a first-line treatment. After 8 weeks of treatment, the patient’s intracranial tumor partially responded. During the treatment, the patient had no other abnormalities (Figure 6).

## Discussion

In recent years, the clinical effect of EGFR-TKIs in the treatment of patients with NSCLC has gradually attracted attention, and several clinical trials have proven their effectiveness (Ballard et al., 2016; Wang et al., 2017; Su et al., 2018; Saida et al., 2019; Yomo et al., 2019; Yun et al., 2019; Dai et al., 2020). A retrospective study (Liu et al., 2020) compared the efficacy of first-generation icotinib and gefitinib against EGFR-mutated NSCLC brain metastases. There was no significant difference in the intracranial overall response rate (ORR) and disease control rate between the groups. The median intracranial progression free survival (PFS) was 8.4 and 10.6 months, respectively, showing that EGFR-TKI treatment is effective. Compared with first-generation TKIs, second-generation TKIs improve the PFS and overall survival (OS) of patients with EGFR-mutant NSCLC, and they are effective in patients with brain metastases. In a comprehensive analysis, afatinib significantly improved PFS, compared with chemotherapy in patients with brain metastases (Schuler et al., 2016). Although the treatment of EGFR-mutant tumors with first- and second-generation TKIs improves tumor control, resistance to first- and second-generation EGFR-TKIs usually occurs within 9–14 months. The EGFR T790M point mutation is observed in more than 50% of patients with disease progression following the use of first-line TKIs (Mok et al., 2009; Maemondo et al., 2010; Mitsudomi et al., 2010; Rosell et al., 2012; Sequist et al., 2013; Wu et al., 2014; Wu et al., 2017). Osimertinib, a third-generation, irreversible EGFR-TKI, inhibits common EGFR mutations as well as the T790M resistance mutation. Its distribution in the brain tissue is higher than that of the earlier generations of TKIs, and the ability to penetrate the BBB is stronger (Ballard et al., 2016). It has superior intracranial performance and anti-tumor activity. However, osimertinib is not ideal in terms of toxicity, which may be related to the active metabolite AZ5104 produced in the body. Almonertinib is a new third-generation EGFR TKI that targets EGFR-sensitizing mutations and T790M resistance mutations. A phase I multicenter clinical trial (NCT0298110) has confirmed that almonertinib is safe, tolerable, and effective in patients with locally advanced or metastatic NSCLC with EGFR T790M mutation (Yang et al., 2020). Compared with osimertinib, almonertinib retains the acrylamide structure, uses aminopyrimidine as the core, and introduces cyclopropyl to increase the stability of the compound (Cross et al., 2014; Uchibori et al., 2017; Dong et al., 2019). After the T790M mutation, the protein forms a compact hydrophobic pocket. The hydrophobic cyclopropyl substituents on the indole and its heteroatoms in the structure can fill this compact hydrophobic pocket and achieve good inhibitory activity and selectivity to WT.

In recent years, the incidence of brain metastases with NSCLC has increased. WBRT is a conventional treatment for brain metastases, and it can alleviate the neurological symptoms of patients with NSCLC brain metastases. However, long-term adverse effects are more significant, and they can cause neurocognitive functional degradation and a decline in the quality of life of patients. Platinum-based chemotherapy drugs are difficult to reach brain tumors due to the BBB, and the median survival time of patients is generally only 3–6 months. The emergence of targeted therapy has transformed the treatment of NSCLC into the era of individualized treatment. With extensive research on NSCLC brain metastasis and the continuous development of molecular targeted drugs for corresponding gene mutations, the patient’s disease control time and overall survival time have been significantly prolonged, bringing hope to patients with NSCLC brain metastasis.

In this study, the administration of almonertinib showed good efficacy in a PC9-LUC nude mouse brain metastasis model. The administration of 25 mg/kg almonertinib led to the regression of brain tumors and improved neurological functions. Consistent with the experimental results, the intracranial lesions in the patient studied were significantly reduced after treatment with almonertinib. The body weight of the nude mice in the osimertinib group decreased more significantly than that of mice in the almonertinib group. The almonertinib group exhibited a longer survival time than the osimertinib group. During the administration period, there was obvious skin keratinization in the face and neck of mice in the 25 mg/kg osimertinib group. We surmise that in the high-dose osimertinib group, the drug may be metabolized into the active compound AZ5104, which has a stronger inhibitory effect on wild-type EGFR, while almonertinib effectively avoids non-selective molecules such as AZ5104 metabolites.

Interestingly, with the method of establishing a brain metastasis model reported by Yun et al. (2019), we found that the nude mice also developed spinal cord metastases when tumor cells were injected at 1 mm below the coronal suture of the skull and 2–2.5 mm from the sagittal suture. Spinal cord metastases can compress normal spinal cord tissue, resulting in the complete loss of the corresponding sphincter function and motor and sensory functions. If not diagnosed and treated on time, this can cause physical disorders and seriously affect the patient’s quality of life. In a previous study, autopsy revealed that approximately 0.9–2.0% of patients with cancer had spinal cord metastasis (Chamberlain et al., 2017), among which lung cancer was the most common primary cancer, accounting for approximately 50% (CHASON et al., 1963). In recent years, EFGR-TKI has achieved good results in the treatment of primary lung adenocarcinoma and brain metastases. However, due to the few cases of intramedullary spinal cord metastasis (ISCM) complicated by lung adenocarcinoma, the effect of targeted therapy is still unclear. Tumor metastasis to the spinal cord is an advanced manifestation of cancer. Approximately 61% of patients also have brain metastases (Sung et al., 2013; Goyal et al., 2019), and 15–55% of patients have metastases to other parts of the body, such as the meninges (Potti et al., 2001; Diehn et al., 2015). However, there are only a few reports on spinal cord metastasis, and the treatment options are relatively limited. In this study, the NSCLC spinal cord metastasis model was established by injecting PC9-LUC cells into different parts of the brains in nude mice. The 10 mg/kg almonertinib treatment group showed a good treatment effect against brain metastases. Therefore, we used 5 and 25 mg/kg almonertinib to treat spinal cord metastatic mice and found that almonertinib can inhibit tumor growth but cannot cause tumor regression when administered at 5 mg/kg. However, 25 mg/kg almonertinib showed a good treatment effect against brain and spinal cord metastases. Consistent with the phenomenon observed in the monotherapy of brain metastases, the almonertinib and osimertinib groups showed similar efficacy.

The BBB can limit the permeation of most molecules, thereby playing the role of a physical barrier (Porteous et al., 2013; Sanchez-Covarrubias et al., 2014). Simultaneously, brain capillary endothelial cells express a variety of pumps that can actively remove exogenous substances that enter the cerebrospinal fluid circulation. However, most chemotherapeutic drugs and available targeted drugs have limited ability to enter the central nervous system through the BBB. In this study, the pharmacokinetic parameters of PC9-LUC brain metastases in mice treated with almonertinib reflected that almonertinib has a higher intracranial concentration and has good BBB penetration. The results of *in vitro* permeation experiments showed that when almonertinib penetrated ABCB1-MDCK and BCRP-MDCK monolayer cells, its effect was influenced by the excretion of ABCB1 and BCRP transporters. The penetration rate of almonertinib at low concentrations is poor, and increasing the dose can significantly increase the penetration rate of almonertinib.

The preclinical study results reported here are promising; almonertinib could be a potential clinically significant treatment option for patients with EGFRm brain and spinal cord metastases. Nonetheless, further investigation of almonertinib in patients with EGFRm NSCLC and brain and spinal cord metastases is warranted.

## Data Availability

The original contributions presented in the study are included in the article/Supplementary Material, further inquiries can be directed to the corresponding authors.
