# Supplementary figures and images for: Experimental Study of Almonertinib Crossing the Blood-Brain Barrier in EGFR-Mutant NSCLC Brain Metastasis and Spinal Cord Metastasis Models
Source: Front Pharmacol. 2021 Sep 24;12:750031. doi: 10.3389/fphar.2021.750031 (PMC8497791; doi:10.3389/fphar.2021.750031)

Actin:
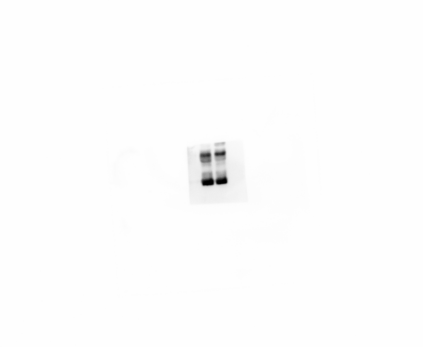

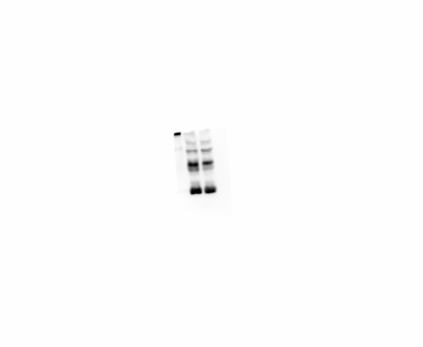


HA:



BCRP:
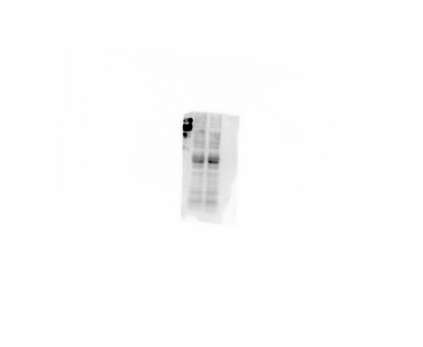


ABCB1:
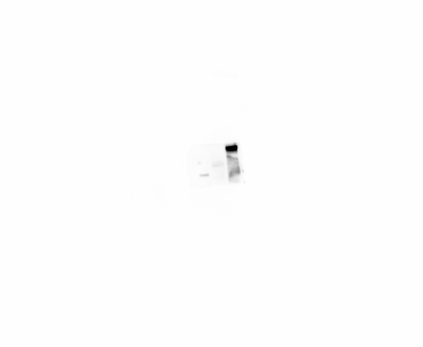


Flag:
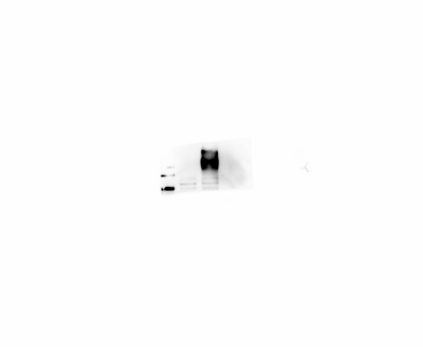

Supplement: Supplementary file 1 [file DataSheet1.docx]
